# Differential effects on neurodevelopment of *FTO* variants in obesity and bipolar disorder suggested by in silico prediction of functional impact: An analysis in Mexican population

**DOI:** 10.1002/brb3.1249

**Published:** 2019-04-29

**Authors:** Erasmo Saucedo‐Uribe, Alma Delia Genis‐Mendoza, Adriana Díaz‐Anzaldúa, José Jaime Martínez‐Magaña, Carlos Alfonso Tovilla‐Zarate, Isela Juárez‐Rojop, Nuria Lanzagorta, Michael Escamilla, Thelma Beatriz González‐Castro, María Lilia López Narvaez, Yazmín Hernández‐Díaz, Humberto Nicolini

**Affiliations:** ^1^ Center of Advanced Neurosciences Department of Psychiatry Autonomous University of Nuevo Leon Hospital Universitario “Dr. José Eleuterio González” Monterrey Mexico; ^2^ Laboratory of Genomics of Psychiatric and Neurodegenerative Diseases National Institute of Genomic Medicine Mexico City Mexico; ^3^ Children's Psychiatric Hospital “Dr. Juan N. Navarro” Psychiatric Attention Services Mexico City Mexico; ^4^ Department of Psychiatric Genetics Clinical Investigations National Institute of Psychiatry Ramón de la Fuente Muñiz Mexico City Mexico; ^5^ Multidisciplinary Academic Division of Comalcalco Universidad Juárez Autónoma de Tabasco Comalcalco Tabasco Mexico; ^6^ Academic Division of Health Sciences Autonomous University of Tabasco Villahermosa Tabasco Mexico; ^7^ Department of Clinical Research Carracci Medical Group Mexico City Mexico; ^8^ Center of Emphasis in Neurosciences Health Sciences Center Texas Tech University El Paso, Texas USA; ^9^ Multidisciplinary Academic Division of Jalpa de Mendez Universidad Juárez Autónoma de Tabasco Comalcalco Tabasco Mexico; ^10^ General Hospital of Yajalón “Manuel Velasco Siles” Ministry of Health of Chiapas Yajalón Chiapas Mexico

**Keywords:** bipolar disorder, *FTO* variants, in silico functional prediction, neurodevelopment

## Abstract

**Introduction:**

Several studies indicate that polygenic obesity is linked to fat‐mass and obesity‐associated (*FTO*) genetic variants. Nevertheless, the link between variants in *FTO* and mental disorders has been barely explored. The present work aims to determine whether *FTO* genetic variants are associated with bipolar disorder and obesity, and to perform an in silico prediction of variant‐dependent functional impact on the developing brain transcriptome.

**Methods:**

Four hundred and forty‐six Mexican mestizos were included in a genetic association analysis. SNP‐sequence kernel association test and linear mixed models were implemented for genetic association assessment. For functional impact prediction, we analyzed the mapping of regulatory elements, the modification of binding sites of transcription factors and the expression of transcription factors in the brain developing transcriptome, searching on different databases.

**Results:**

In the set‐based analysis, we found different associated regions to BD (bipolar disorder) and obesity. The promoter flanking region of *FTO* intron 1 was associated with differential effects on BMI, while intron 2 of *RPGRIP1L* and *FTO* upstream regions were associated with BD. The prediction analysis showed that *FTO* BD‐associated variants disturb binding sites of *SP1* and *SP2;* obesity‐associated variants, on the other hand, disturb binding sites of *FOXP1,* which are transcription factors highly expressed during prenatal development stages of the brain.

**Conclusion:**

Our results suggest a possible effect of *FTO* variants on neurodevelopment in obesity and bipolar disorder, which gives new insights into the molecular mechanism underlying this association.

## 1 INTRODUCTION

One of the strongest associations to obesity was established in 2007, when in a genome‐wide association study (GWAS) for type 2 diabetes, common variants of the *FTO* gene showed an association with this trait, but subsequent analyses revealed that this association was modulated by body mass index (BMI) (Frayling et al., 2007; Scuteri et al., 2007). The relation between *FTO* common variants and obesity has been replicated in diverse populations worldwide, including Mexican population (Andreasen et al., 2008; Dina et al., 2007; Do et al., 2008; Grant et al., 2008; Saldana‐Alvarez et al., 2016; Villalobos‐Comparan et al., 2008).

Obesity‐related illnesses, such as cardiovascular diseases (CVD), have been established as leading causes of death in individuals with bipolar disorder (BD) (Goldstein et al., 2011; Osby, Brandt, Correia, Ekbom, & Sparen, 2001; Weiner, Warren, & Fiedorowicz, 2011). It is suggested that some drugs used for treating individuals with BD cause weight gain, which has been settled as one possible explanation of the frequent comorbidity between CVD risk, obesity, and BD (Keck et al., 2007; Shah, Shen, & El‐Mallakh, 2006). A recent study in Mexican individuals diagnosed with BD suggests that common *FTO* variants could modulate BMI in these patients and there could be a possible interaction between mood‐stabilizers (Diaz‐Anzaldua et al., 2015). Nevertheless, a high prevalence of overweight has been reported in some treatment‐naïve patients, which suggests that there are other factors involved in this relationship (Maina, Salvi, Vitalucci, D'Ambrosio, & Bogetto, 2008). In these sense, some studies have reported that BD patients have a diminished ability to modulate self‐mood by over‐eating (Elmslie, Mann, Silverstone, Williams, & Romans, 2001; Fleet‐Michaliszyn et al., 2008; Wildes, Marcus, & Fagiolini, 2006). Supporting the evidence that a *FTO* genetic variation could have an effect on BMI modulation by mood‐state regulation in patients with BD, an association with *FTO* variants has also been found in patients with major depressive disorder and eating‐disorders, (Castellini et al., 2017; Hung et al., 2014; Muller et al., 2012). Therefore, we decided to evaluate the association of *FTO* polymorphisms with bipolar disorder and obesity using aggregate variation; additionally, we also performed an in silico prediction analysis of associated variants to search for their possible functional impact on developing brain transcriptome.

## 2 MATERIAL AND METHODS

### 2.1 Study population

We included a total of 446 Mexican mestizo individuals. The sample was divided into two groups based on their psychiatric diagnosis. The first group included 276 individual diagnosed with bipolar disorder, of whom 176 were female (63.77%) and 100 were male (36.23%) based on the DSM‐IVR (Rabe‐Jablonska & Bienkiewicz, 1994). The mean age was 37.57 ± 13.85. BD patients were recruited from two medical centers in Mexico City, National Institute of Psychiatry Ramón de la Fuente Muñiz, these patients had been previously included in other publications (Diaz‐Anzaldua et al., 2015) and the Group of Medical and Family Studies Carracci. Of the 276 individuals diagnosed with BD, 129 were recruited from the National Institute of Psychiatry Ramón de la Fuente Muñiz and 147 from the Group of Medical and Family Studies Carracci; all individuals were diagnosed between 2010 and 2014. The second group included 170 individuals (126 were female (74.12%) and 44 (25.88%) were male) who did not have any psychiatric pathology and were considered as controls (BDC), they were recruited from the Group of Medical and Family Studies Carracci. The mean age was 38.91 ± 13.27. Every individual included in this study was administered a structured questionnaire to collect sociodemographic data (Table 1).

**Table 1:** Descriptive characteristics of the population

Characteristics	BD *n* = 276	BDC *n* = 170	χ^2^/F	*p* value^b^
Age, years ± SD	37.57 (13.85)	38.97 (13.27)	0.2851	0.78
Gender				
Male (%)	100 (36.23)	44 (25.88)	4.6917	0.03
Female (%)	176 (63.77)	126 (74.12)		
Body mass index, BMI, (kg/m^2)^	28.25 (5.78)	28.56 (5.78)	1.0986	0.27
Normal weight (%)^a^	89 (32.25)	54 (31.76)	6.7729	0.03
Overweight (%)	114 (41.31)	53 (31.18)		
Obese (%)	73 (26.45)	63 (37.06)		

BD: bipolar disease subjects; BDC: bipolar disease controls.

a The sample was stratified based on BMI into three groups: Normal weight < 25.00 kg/m^2^, Overweight 25.00–29.99 kg/m^2^ and Obese > 30.00 kg/m^2^.

b For categorical variables a χ^2^ test, and for continuous variables a Student′s *t* test was performed.

### 2.2 Ethical statement

In an interview, the participants were informed about the aims of the study and signed a written‐informed consent. The protocol was approved by the Investigation and Ethics Committees of the National Institute of Genomic Medicine in Mexico (INMEGEN, Approval number: 20/2014/I).

### 2.3 Genotyping and imputation of missing variants

DNA was isolated from peripheral leukocytes, then it was submitted to direct genotyping of seven single‐nucleotide polymorphisms (SNP's) using TaqMan assays (Applied Biosystems, Foster City, CA), with primers and conditions according to the manufacturer's protocol. The seven variants are included in a region that spans a total of 91 kb in the *FTO* region (rs6499640, rs9939973, rs1421085, rs1558902, rs8050136, rs9939609 and rs8061518). We selected these variants for direct genotyping because they have been previously associated to modulation of BMI in different populations, including the Mexican population (Chauhan et al., 2011; Ehrlich & Friedenberg, 2016; Park et al., 2013; Saldana‐Alvarez et al., 2016; Solak et al., 2014; Villalobos‐Comparan et al., 2008; Wang et al., 2013; Wu et al., 2014).

All missing genotypes were imputed, spanning a genomic region on chromosome 16 (including the last introns of *RPGRIP1L* and the first introns of *FTO*) that started on 53700000 bp and ended on 53900000 bp from the p telomere (based on the hg19 coordinates) using Beagle software and the 1000 Genomes Phase 3 as reference panel (Auton et al., 2015; Browning & Browning, 2016). In order to establish the phasing of the input data, Beagle performed a phasing of the introduced unphased genotypes prior to the imputation process; it used the phase reference panel just as previously described by Browning & Browning (2007).

For the statistical analysis, we only considered variants with a MAF > 1% and a *p* value > 0.0001 for a χ2 test for Hardy–Weinberger equilibrium. After imputation, we obtained a total of 1,422 variants; nevertheless, due to the previously established criteria, only 340 variants remained and were included in the following analysis.

### 2.4 Genetic association tests

For the genetic association of genetic variants with BD and BMI phenotype, we performed two different approaches: (a) a set‐based association test, with variants grouped by the mapping of regulatory and coding sequences to the gene; and (b) a single‐variant analysis, implementing a linear mixed model. For the set‐based association test, the SNP sequence kernel association test (SKAT) was implemented in R (RRID:SCR_001905); also, linear mixed models were implemented on the genome‐wide complex trait analysis (GCTA) software to analyze single‐variants (Yang, Lee, Goddard, & Visscher, 2011; Wu et al., 2011). The BD phenotype was coded as a categorical variable, BMI was considered a continuous variable expressed in kg/m^2.^, obesity criteria followed previous reports (Hubry & Hu, 2015). A SKAT and mixed model *p* < 0.05 was considered statistically significant after adjustment for age, gender and genetic relationship matrix (GRM), as well as after FDR correction for multiple testing (i.e. throughout the text *p*‐values are reported after FDR multiple testing correction). The GRM was calculated based on the algorithm implemented using the efficient mixed‐model association expedited software (EMMAX) and all imputed variants (Zhou & Stephens, 2012). Imputed variants that showed a positive association were validated through Sanger sequencing in 20% of the samples.

### 2.5 Prediction of functional effects

The functional impact of variants was predicted using Variant Effect Predictor software (VEP) (RRID:SCR_007931), Encyclopedia of DNA elements (ENCODE) (RRID:SCR_006793) and SNP2TFBS database (RRID:SCR_016885) (“An integrated encyclopedia of DNA elements in the human genome, 2012; Kumar, Ambrosini, & Bucher, 2017; McLaren et al., 2016). We used the SNP2TFBS database to explore if previous phenotype‐associated variants could disrupt a binding site of transcription factors (TF). The SNP2TFBS is a collection of binding scores reported for the collection of variants with a minor allele frequency, higher than 1% in the 1000 genomes project (Auton et al., 2015) based on the position weight matrix generated by JASPAR (RRID:SCR_003030) (Sandelin, Alkema, Engstrom, Wasserman, & Lenhard, 2004) to different TF. In this database, one can search the “rs” identifier of a particular variant and the database reports the TF binding sites that could be disrupted by this variant. Once we generated the list of variant‐TF disrupted binding‐site pairs, we checked if this TF could have an impact on the neurodevelopment transcriptome. The analysis of transcription factors expressed in brain during different neurodevelopmental stages was performed using the query of developmental transcriptome reported in the BrainSpan database (RRID:SCR_008083) (Tebbenkamp, Willsey, State, & Sestan, 2014). The BrainSpan database is a public database, data of coding and non‐coding transcripts of the human brain generated by different technologies are available to download and can be used in further analyses. In this database, we found the transcript levels at differential development stages of the human brain (from prenatal stages to adulthood) measured in different cerebral areas. Using BrainSpan we explored transcription factors that showed sites disrupted by associated variants which are highly expressed in the prenatal brain and scarcely expressed in postnatal brain.

## 3 RESULTS

On average, individuals of the BD group were younger than those of the BDC group, but this difference did not reach statistical significance. The overview descriptive characteristics of the sample are shown in Table 1. Regarding the mean BMI, there were no significant differences between the two groups; however, after BMI stratification, the BD group showed a higher prevalence of overweight than the BDC; while the BDC group showed a higher frequency of obesity (chi‐square test, *p* < 0.05).

### 3.1 Differential association of variants in FTO between obesity and bipolar disorder

Set‐based association analysis with BD as the phenotype trait revealed that two regions reached a statistically significant association, intron 2 of *RPGRIP1L* and the *FTO* upstream region. A summary of the set‐based association analysis is reported in Table 2. When we considered BMI as a continuous variable for the whole sample (BD and BDC groups), variants in intron 1 promoter flanking of *FTO* reached statistical significance (*p* < 0.05) and after diagnosis stratification this association remained only in the BD group (*p* < 0.05), even when the BDC group had a higher rate of obesity.

**Table 2:** Association results for set‐based analysis

Gene	Predicted effect	Number of Variants	BD(*p* value)^a^	BMI(*p* value)^b^
*RPGRIP1L*	Intron 6	4	0.2657	**0.0750**
	Intron 2	3	**0.0422** c	0.2128
*FTO*	Upstream	2	**0.0422**	0.2133
	Intron 1	187	0.9659	**0.0656**
	Intron 1 Open chromatin	5	1.0000	0.5242
	Intron 1 Promoter flanking	5	0.7891	**0.0453** d
	Intron 1 TF binding site	4	1.0000	0.2190
	Intron 2	23	0.5725	0.5940
	Intron 2 Enhancer	2	0.4013	0.5316
	Intron 3	55	0.6995	0.7831
	Intron 4	50	0.9435	0.2568

a *p* value for the bipolar disease association analysis.

b *p* value for body mass index (BMI) association analysis.

c In bold are the significant *p*‐values or the sets that reached statistical significance (*p* < 0.05, *p*‐values are reported after FDR correction). The set‐based aggregation was performed based on the in silico predicted effect of the variants.

d Only this region reached statistical significance after the inclusion of BD in the models (*FTO*‐Intron1 : *p* = 0.0402).

The analysis of single‐variants revealed that seven variants reached a statistically significant association with BD (*p* < 0.05); these BD‐associated variants were mapped to intron 2 of *RPGRIP1L*,* FTO* upstream region and intron 1 promoter flanking of *FTO*. When we considered obesity (BMI > 30 kg/m^2^) as phenotype, 14 variants in the promoter flanking region immersed in *FTO* intron 1 reached a statistically significant association (*p* < 0.05). Rs7205859, in the promoter flanking region immersed in intron 1 of *FTO*, was the only variant associated to BD and obesity (*p* < 0.05).

### 3.2 Prediction of functional impact (“in silico”) of variants in the developing brain

**Figure 1:**
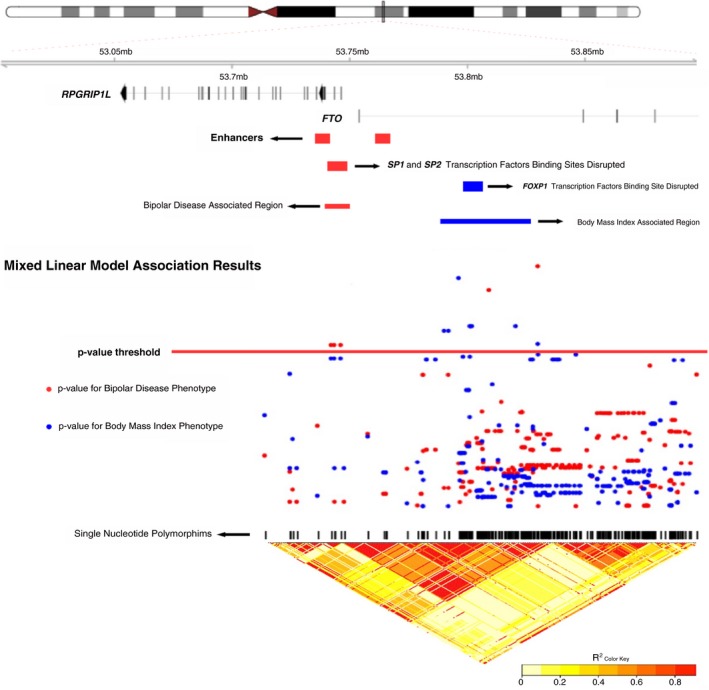
Summary of the in silico predicted functional analysis

In order to correlate associated variants with expression‐regulatory elements that could alter brain development, we performed a query of the associated variants to different databases. After mapping the associated variants with expression‐regulatory elements in DNA, we found that BD‐associated variants are in high linkage disequilibrium with an enhancer located in intron 1 of *RPGRIP1L*; while obesity‐associated variants are in high linkage disequilibrium with an enhancer located in intron 1 of *FTO*, suggesting a possible differential role of *FTO* variants in BD and obesity. The inclusion of genomic information at different levels is summarized in Figure 1. We also mapped variants that could modulate binding sites of transcription factors with a query to SNP2TFBS database. BD‐associated variants could disturb binding sites of *SP1, SP2, IRF1, PAX4* and *RUNX2;* whereas obesity‐associated variants could disturb binding sites of *IRF1, PAX5, ARID3A, KLF1, KLF4, KLF5* and *FOXP1*. The description of variants that disrupt binding sites of the previous TF is shown in Table 3, including alleles and the identifier of variants. Additionally, we searched for transcription factors that could modulate neurodevelopment; we filtered only transcription factors highly expressed in the brain during prenatal brain stages, with a query to the BrainSpan database: *SP1* and *SP2* for BD‐associated variants and *FOXP1* for obesity‐associated variants. The mapping expression of these transcription factors revealed that *SP1* and *SP2* are highly expressed in the ganglionic eminence, while *FOXP1* is highly expressed in the striatum brain zone at prenatal stages.

**Table 3:** Report of variants that disrupted a transcription factor binding site

Chromosome	Genomic position	Genetic Variant	Alleles	Transcription factor binding site disrupted
Bipolar disease‐associated variants				
16	53732455	rs182423166	A/G	SP1/SP2
	53825999	rs7205859	A/T	IRF1
	53839354	rs576360210	GT/G	PAX4/RUNX2
Obesity‐associated variants				
16	53715308	rs4310844^a^	C/T	ARID3A
	53779568	rs11380777	T/TC	PAX5
	53789612	rs1108103	G/T	KLF1/KLF4/KLF5
	53815864	rs8054237^a^	G/A	FOXP1
	53825999	rs7205859	A/T	IRF1

a Variant previously associated to obesity (Hassanein et al., 2010; Li et al., 2013).

## 4 DISCUSSION

*FTO* is one of the genes known as a post‐GWAS gene, being mainly associated with obesity. Obesity is a complex process in which different systems and organs participate, including the brain. In recent studies, it has been hypothesized that genetic variants affecting brain development could have a high impact on obesity, establishing a possible link between molecular mechanisms modulating obesity and brain disorders (Hara et al., 2001; McCammon, Blaker‐Lee, Chen, & Sive, 2017; Yeo & Heisler, 2012). In this study, we analyzed a genetic association of *FTO* variants with BD and obesity using a variant aggregation strategy, and we also performed an in silico prediction analysis of the possible functional impact of these variants in the developing brain.

There is a known hypothesis that explains the high obesity rates in patients with BD, which relates to the use of drugs related to weight gain in treatment schemes of these patients (Keck et al., 2007; Shah et al., 2006). Nevertheless, another hypothesis supported by the fact that naïve treatment patients have higher rates of obesity than the general population, established that the link between BD and obesity could also be explained by a disrupted brain biological process in individuals with BD (McElroy & Keck, 2012). In this line of evidence, we found that *FTO* variants associated with BMI only remained in BD patients, even when the BDC group had higher rates of obesity, which suggests that the effect of these variants modulating BMI could be higher in BD patients than in non‐BD obese individuals. Supporting this result, a neuro‐image study recently reported that carriers of *FTO*‐obesity risk variants have a reduced longitudinal functioning in the medial prefrontal cortex and this reduction correlated with higher rates of impulsivity, behavioral disinhibition, and risky decision‐making, which are common symptoms in BD (Chuang et al., 2015). These *FTO*‐associated changes in brain‐behavior relationship lead us to hypothesize that the link between BD symptomatology and eating behavior (particularly the way in which eating is controlled) could be a process modulated by *FTO* genetic variants. However, we did not search for eating‐related endophenotypes that could lead us to more precise *FTO*‐associated behavioral changes in BD patients.

The molecular mechanism underling the association of *FTO* variants with obesity remains largely unrevealed; some in vivo and in vitro studies applying different molecular strategies have returned inconclusive results. The FTO protein is an Alk‐B like DNA/RNA demethylase, *FTO* knock‐out mice survive until adulthood stages and exhibit no differential weight changes when compared to wild‐type mice, suggesting that alterations in protein structure do not indicate a risk of obesity (Han et al., 2010; Ronkainen et al., 2015; Stratigopoulos et al., 2008). One of the most accepted hypotheses to explain the molecular mechanism takes into consideration that the expression regulation of *FTO* and other nearby genes (*RPGRIP1L* and *IRX3*) is a process controlled by an obesity‐associated region in intron 1 of *FTO*, which we also replicated in this study (Ronkainen et al., 2015; Smemo et al., 2014; Stratigopoulos et al., 2014). Nevertheless, divergent results in animal models have been reported. Some results indicate a possible dysregulation of *IRX3* whereas others point to *RPGRIP1L*. The lack of consistency among reports may be due to the use of dissimilar stages of tissue development when analyzing gene expression. The brain is a complex organ which has differential spatial‐temporal gene expression patterns depending on the developmental stage, this differential pattern could be a key regulatory factor during the wiring development process of this organ (Chedotal & Richards, 2010; Chou et al., 2016; French, Tan, & Pavlidis, 2011). In the present analysis, we found a different variant association pattern when we considered BD or obesity as a phenotype trait, which suggests differential modulating effects of these variants in these diseases. In an attempt to yield new insights of a possible mechanism underlying the association of *FTO*‐obesity risk variants with BD, we performed an in silico prediction algorithm consulting different databases. The in silico prediction analysis showed that *FTO‐*risk variants could have a direct effect on modulating binding sites of transcription factors such as *SP1*,* SP2* and *FOXP1* during brain development, which suggests a possible effect of these transcription factors on a *FTO*‐risk variant dependent on brain development mechanism. Supporting these data, in animal models it has been described that alterations in expression levels of these transcription factors (*SP1*,* SP2* and *FOXP1*) have a direct effect reducing the morphogenesis of the brain (Li et al., 2015; Valin, Cook, Ross, Saklad, & Gill, 2009). Binding sites of *SP1* and *SP2* were predicted to be disturbed by BD‐associated variants, and are transcription factors that play critical roles in embryonic and early development (Safe, Imanirad, Sreevalsan, Nair, & Jutooru, 2014). SP transcription factors has been implicated in cell differentiation and the fate of superficial cortical interneurons of cells that derivate from ganglionic eminence, suggesting that this transcription factors could be linked with the neuropathology of psychiatric disorders (Liu et al., 2018; Ma et al., 2012; Miyoshi et al., 2010). Reinforcing this hypothesis, previous reports have associated brain transcripts levels alterations on this TF in patients diagnosed with BD, schizophrenia and other mood‐related disorders (Ben‐Shachar & Karry, 2007; Pinacho et al., 2011; Shi et al., 2011; Shyn et al., 2011; Tam et al., 2010). Analyzing *FOXP1* (variants associated with obesity could disturb the binding site of this TF) has been previously associated with speech development, and deleterious genetic variants are known to cause FOXP1 syndrome (Siper et al., 2017); patients with this syndrome have psychiatric alterations and almost 30% of them are obese (Le Fevre et al., 2013; Lloveras et al., 2014), suggesting a possible link between *FOXP1* and obesity. *FOXP1* is a TF critical in the fate of adult striatum medium spiny neurons projection and its transcript is highly up‐regulated in the whole ganglionic eminence (Precious et al., 2016). A possible link between striatum and obesity has been found in a neuroimaging study using positron emission tomography, and reported that the striatum was the most altered in diet‐induced obesity, which reinforces a possible link between the brain and obesity (Michaelides et al., 2018). Notwithstanding, more studies should be performed to achieve a conclusive explanation of the relationship between these transcription factors and *FTO*‐risk variants during the dynamics of brain development.

One of the limitations of the present work is that patients analyzed were not drug‐naïve which could be a confounding factor in this population; nevertheless, this work reports an association of *FTO* variants with bipolar disorder. To the best of our knowledge, this is the first study reporting differential *FTO*‐risk for obesity and bipolar disorder. Also, we performed a prediction analysis of the regulatory function of these *FTO*‐risk variants, to gain new insight into the molecular functioning of this region in the developing brain. However, it is necessary to perform more functional in vivo and in vitro analyses to identify the mechanism underling these associations.

## 5 CONCLUSION

The present work found an association of *FTO* variants with bipolar disorder. Also, we performed a prediction analysis of the regulatory function of these *FTO*‐risk variants, to give new insights into the molecular functioning of this region in the developing brain.

## CONFLICT OF INTERESTS

The authors declared no conflict of interest.
